# New Sesquiterpenoids and Anti-Platelet Aggregation Constituents from the Rhizomes of *Curcuma zedoaria*

**DOI:** 10.3390/molecules21101385

**Published:** 2016-10-17

**Authors:** Jih-Jung Chen, Tung-Han Tsai, Hsiang-Ruei Liao, Li-Chai Chen, Yueh-Hsiung Kuo, Ping-Jyun Sung, Chun-Lin Chen, Chun-Sheng Wei

**Affiliations:** 1 Department of Pharmacy, Tajen University, Pingtung 907, Taiwan; jjc5711@yahoo.com.tw (T.-H.T.); icupdrab@gmail.com (L.-C.C.); 2 Department of Medical Research, China Medical University Hospital, China Medical University, Taichung 404, Taiwan; 3 Graduate Institute of Natural Products, College of Medicine, Chang Gung University, Taoyuan 333, Taiwan; liaoch@mail.cgu.edu.tw; 4 Department of Pharmacy, Zuoying Branch of Kaohsiung Armed Forces General Hospital, Kaohsiung 813, Taiwan; pharmacist@ngh.com.tw; 5 Department of Chinese Pharmaceutical Sciences and Chinese Medicine Resources, China Medical University, Taichung 404, Taiwan; kuoyh@mail.cmu.edu.tw; 6 Department of Biotechnology, Asia University, Taichung 413, Taiwan; 7 National Museum of Marine Biology and Aquarium, Pingtung 944, Taiwan; pjsung@nmmba.gov.tw; 8 Department of Biological Sciences, National Sun Yat-sen University, Kaohsiung 804, Taiwan; chunlinchen@mail.nsysu.edu.tw

**Keywords:** *Curcuma zedoaria*, Zingiberaceae, rhizomes, sesquiterpenoids, anti-platelet aggregation

## Abstract

Two new sesquiterpenoids—13-hydroxycurzerenone (**1**) and 1-oxocurzerenone (**2**)—have been isolated from the rhizomes of *Curcuma zedoaria*, together with 13 known compounds (**3**–**15**). The structures of two new compounds were determined through spectroscopic and MS analyses. Among the isolated compounds, 13-hydroxycurzerenone (**1**), 1-oxocurzerenone (**2**), curzerenone (**3**), germacrone (**4**), curcolone (**5**), procurcumenol (**6**), ermanin (**7**), curcumin (**8**), and a mixture of stigmast-4-en-3,6-dione (**12**) and stigmasta-4,22-dien-3,6-dione (**13**) exhibited inhibition (with inhibition % in the range of 21.28%–67.58%) against collagen-induced platelet aggregation at 100 μM. Compounds **1**, **5**, **7**, **8**, and the mixture of **12** and **13** inhibited arachidonic acid (AA)-induced platelet aggregation at 100 μM with inhibition % in the range of 23.44%–95.36%.

## 1. Introduction

*Curcuma zedoaria* (Christm.) Rosc. (Zingiberaceae) is a perennial rhizomatous herb indigenous to Bangladesh, Sri Lanka, and India, and is also widely cultivated in China, Japan, Brazil, Nepal, and Thailand. Various sesquiterpenoids [1,2,3,4], curcuminoids [3], benzenoids [3], and their derivatives were isolated from this plant in previous studies. Many of these compounds exhibit anti-inflammatory [1], anti-babesial [2], cytotoxic [3], and anti-fungal [4] activities.

**Figure 1:**
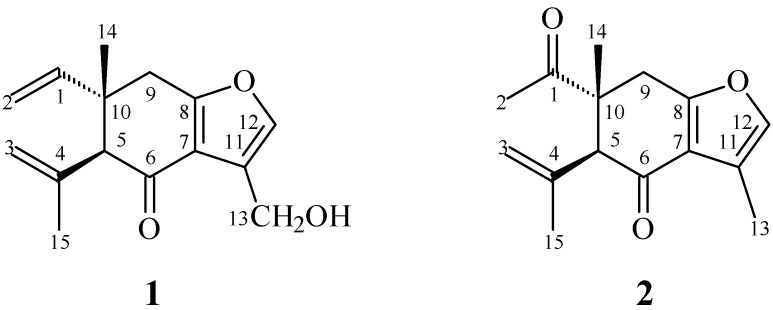
The chemical structures of new compounds **1** and **2** isolated from *C. zedoaria*.

**Figure 2:**
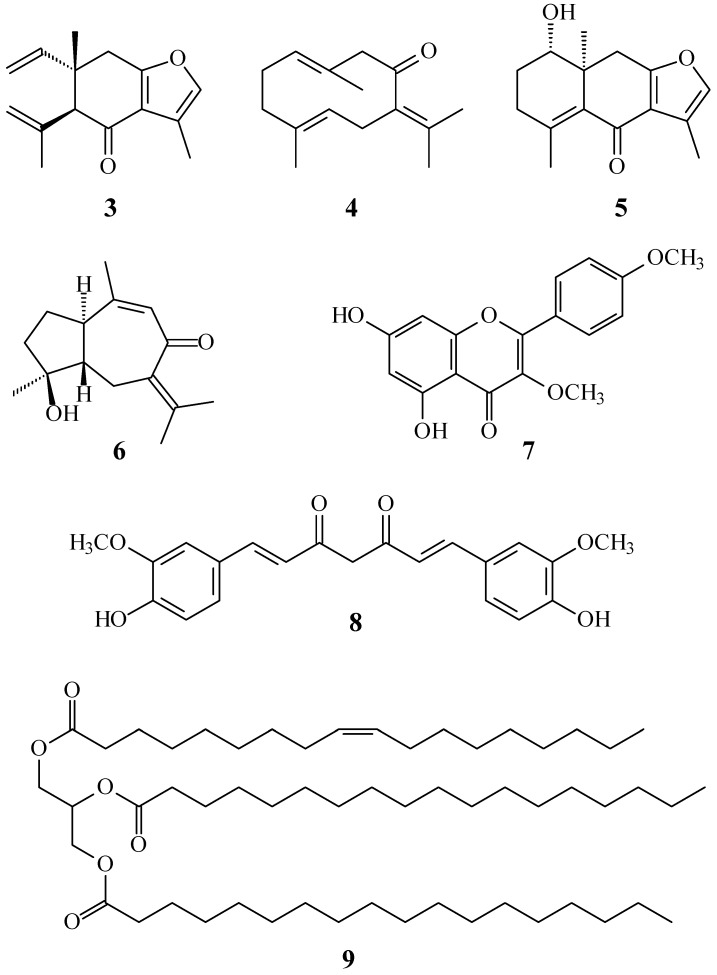

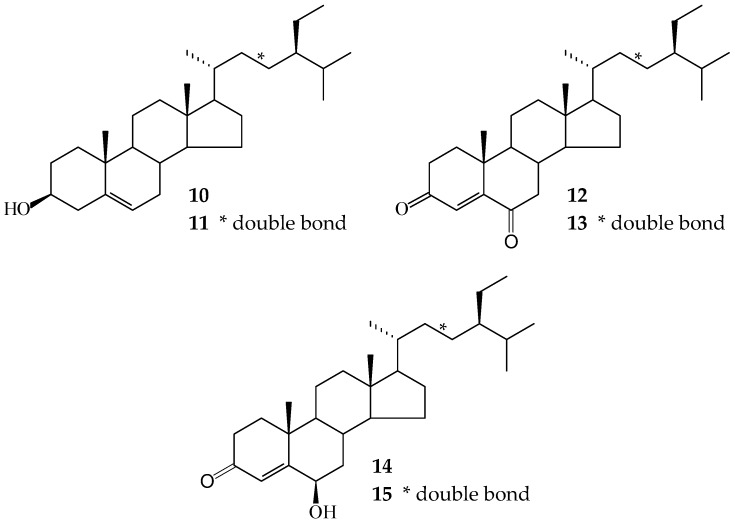
The chemical structures of known compounds **3**–**15** isolated from *C. zedoaria*.

In our studies of medicinal plants for in vitro anti-platelet aggregation activity, *C. zedoaria* was found to be an active species. The MeOH extract of the rhizomes of *C. zedoaria* displayed antiplatelet aggregation activity. Figure 1 illustrates the structures of two new sesquiterpenoids, 13-hydroxycurzerenone (**1**) and 1-oxocurzerenone (**2**). Thirteen known compounds (**3**–**15**) have been isolated and identified from the rhizomes of *C. zedoaria,* and their structures are depicted in Figure 2. This paper describes the structural elucidation of new compounds **1** and **2** and the anti-platelet aggregation activities of the isolates.

## 2. Results and Discussion

**Figure 3:**
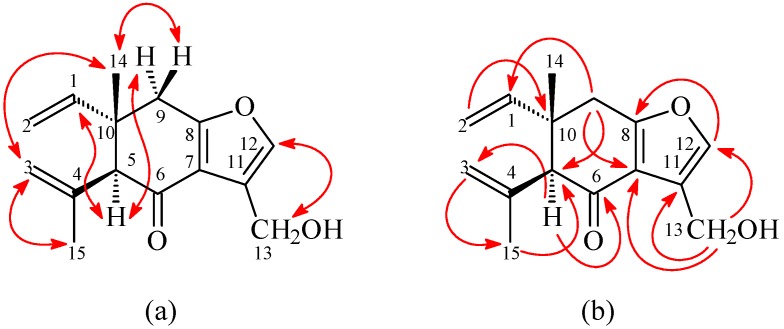
Key (**a**) NOESY and (**b**) HMBC correlations of **1**.

13-Hydroxycurzerenone (**1**) was isolated as a yellowish oil. Its molecular formula (C_15_H_18_O_3_) was determined on the basis of the *quasi*-molecular ion at *m*/*z* 269.1157 ([M + Na]^+^, calcd. for C_15_H_18_O_3_Na: 269.1154) in the high-resolution electrospray ionization mass spectrometry (HR-ESI-MS) spectrum (positive-ion mode) and was supported by the ^1^H-, ^13^C-, and DEPT NMR data. The presence of a conjugated carbonyl group was revealed by the band at 1678 cm^−1^ in the IR spectrum, which was confirmed by the resonance at δ_C_ 194.0 in the ^13^C-NMR spectrum. The IR of **1** also showed OH absorption at 3450 cm^−1^. The ^1^H-NMR spectrum of **1** showed resonances for an aromatic proton (δ_H_ 7.09 (1H, s, H-12)), a hydroxymethyl group (δ_H_ 4.86 (2H, s, H-13)), a methyl group (δ_H_ 1.19 (3H, s, H-14)), a vinyl group (δ_H_ 4.96 (1H, d, *J* = 17.5 Hz, H-2), 4.97 (1H, d, *J* = 11.0 Hz, H-2), 5.82 (1H, dd, *J* = 17.5, 11.0 Hz, H-1)), a prop-1-en-2-yl group (δ_H_ 1.84 (3H, s, H-15), 4.76 (1H, s, H-3), 5.01 (1H, s, H-3)), a methine proton (δ_H_ 3.02 (1H, s, H-5)), and two methylene protons (δ 2.79 (1H, d, *J* = 17.5 Hz, H-9), 2.91 (1H, d, *J* = 17.5 Hz, H-9)). Comparison of the ^1^H- and ^13^C-NMR data (Table 1) of **1** with those of curzerenone (**3**) [5,6] suggested that their structures are closely related, except that the 11-hydroxymethyl group [δ_H_ 4.86 (2H, s, H-13); δ_C_ 52.2 (C-13)] of **1** replaced the 11-methyl group of curzerenone (**3**) [5,6]. This was supported by HMBC correlation between H-13 (δ_H_ 4.86) and C-7 (δ_C_ 120.2), C-11 (δ_C_ 116.0), and C-12 (δ_C_ 139.6), and NOESY correlations between H-13 (δ_H_ 4.86) and H-12 (δ_H_ 7.09). The NOESY cross-peaks (Figure 3) between H-1 (δ_H_ 5.82)/H-5 (δ_H_ 3.02), H-1/H_α_-9 (δ_H_ 2.79), H-14 (δ_H_ 1.19)/H_a_-3 (δ_H_ 5.01), and H-14/H_β_-9 (δ_H_ 2.91) suggested that the 10-vinyl group, H-5, and H_α_-9 are α-oriented, and the 10-methyl group, C-5 prop-1-en-2-yl group, and H_β_-9 are β-oriented. The full assignments of ^1^H- and ^13^C-NMR resonances were supported by ^1^H–^1^H COSY, DEPT, HSQC, NOESY (Figure 3), and HMBC (Figure 3) spectral analyses. On the basis of the above data, the structure of **1** was elucidated as 13-hydroxy-curzerenone.

**Figure 4:**
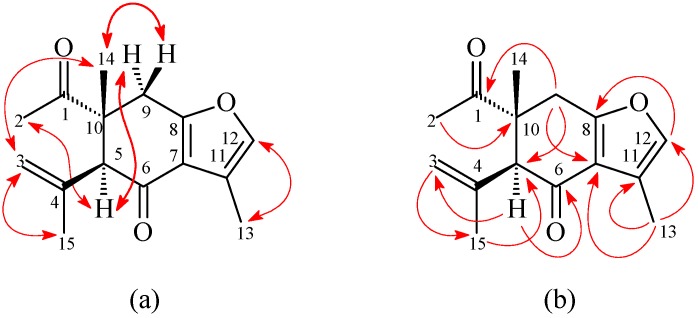
Key (**a**) NOESY and (**b**) HMBC correlations of **2**.

1-Oxocurzerenone (**2**) was isolated as a yellowish oil. The ESI-MS afforded the *quasi*-molecular ion [M + Na]^+^ at *m*/*z* 269, implying a molecular formula of C_15_H_18_O_3_Na, which was confirmed by the HR-ESI-MS (*m*/*z* 269.1153 [M + Na]^+^, calcd. 269.1154). The presence of two carbonyl groups was revealed by the bands at 1675 and 1706 cm^−1^ in the IR spectrum, and was confirmed by the resonances at δ_C_ 194.0 and 212.1 in the ^13^C-NMR spectrum. The ^1^H- and ^13^C-NMR data (Table 1) of **2** were similar to those of curzerenone (**3**) [5,6], except that the 10-acetyl group (δ_H_ 2.13 (3H, s, H-2); δ_C_ 25.6 (C-2), 212.1 (C-1)) of **2** replaced the 10-vinyl group of curzerenone (**3**) [5,6]. This was supported by HMBC correlations between H-2 (δ_H_ 2.13) and C-1 (δ_C_ 212.1) and C-10 (δ_C_ 46.2), and NOESY correlations between H-2 (δ_H_ 2.13) and H-5 (δ_H_ 3.22) and H_α_-9 (δ_H_ 2.94). The NOESY cross-peaks (Figure 4) between H-2 (δ_H_ 2.13)/H-5 (δ_H_ 3.22), H-2/H_α_-9 (δ_H_ 2.94), H-14 (δ_H_ 1.18)/H-3 (δ_H_ 5.01), and H-14/H_β_-9 (δ_H_ 3.06) suggested that the 10-acetyl group, H-5, and H_α_-9 are α-oriented, and the 10-methyl group, C-5 prop-1-en-2-yl group, and H_β_-9 are β-oriented. According to the above data, the structure of **2** was elucidated as 1-oxocurzerenone. This was further confirmed by the ^1^H–^1^H-COSY, NOESY (Figure 4), DEPT, HSQC, and HMBC (Figure 4) techniques.

The known isolates were readily identified by a comparison of physical and spectroscopic data (UV, IR, ^1^H-NMR, [α]_D_, and MS) with corresponding authentic samples or literature values, and this included four sesquiterpenoids, curzerenone (**3**) [5,6], germacrone (**4**) [7], curcolone (**5**) [8], and procurcumenol (**6**) [9], a flavonoid, ermanin (**7**) [10], a curcuminoid, curcumin (**8**) [11], a triacylglycerol, 1-oleoyl-2,3-distearoylglycerol (**9**) [12,13], and six steroids, a mixture of β-sitosterol (**10**) and stigmasterol (**11**) [14,15], a mixture of stigmast-4-en-3,6-dione (**12**) and stigmasta-4,22-dien-3,6-dione (**13**) [16], and a mixture of 6β-hydroxystigmast-4-en-3-one (**14**) and 6β-hydroxystigmasta-4,22-dien-3-one (**15**) [17,18].

The anti-platelet effects of the isolates from the rhizomes of *C. zedoaria* were tested in vitro using the turbidimetric method [19] in washed rabbit platelets induced by collagen (10 μg/mL) and arachidonic acid (AA, 200 μM). The anti-platelet aggregation data are shown in Table 2. The clinically applied anti-platelet agent, aspirin, was used as the positive control. From the results of our anti-platelet aggregation tests, the following conclusions can be drawn regarding these isolates: (a) 13-Hydroxycurzerenone (**1**), 1-oxocurzerenone (**2**), curzerenone (**3**), germacrone (**4**), curcolone (**5**), procurcumenol (**6**), ermanin (**7**), curcumin (**8**), and a mixture of stigmast-4-en-3,6-dione (**12**) and stigmasta-4,22-dien-3,6-dione (**13**) exhibited inhibition (with inhibition % in the range of 21.28–67.58) against collagen-induced platelet aggregation at 100 μM; (b) compounds **1**, **5**, **7**, **8**, and the mixture of **12** and **13** inhibited arachidonic acid (AA)-induced platelet aggregation at 100 μM with inhibition % in the range of 23.44–95.36; (c) ermanin (**7**) was the most effective among the isolates, with inhibition % of 67.58 ± 3.82 against collagen-induced platelet aggregation at 100 μM; (d) curcumin (**8**) showed the strongest anti-platelet aggregation activity induced by AA with inhibition % of 95.36 ± 1.58; (e) our study suggests that *C. zedoaria* and its isolates (especially **3**, **7**, and **8**) could be further developed as potential candidates for the treatment or prevention of cardiovascular diseases.

## 3. Materials and Methods

### 3.1. General Experimental Procedures

Melting points were determined on a Yanaco micro-melting point apparatus and were uncorrected. Optical rotations were measured using a Jasco DIP-370 polarimeter (Japan Spectroscopic Corporation, Tokyo, Japan) in CHCl_3_. Ultraviolet (UV) spectra were obtained on a Jasco UV-240 spectrophotometer. Infrared (IR) spectra (neat or KBr) were recorded on a Perkin Elmer 2000 FT-IR spectrometer (Perkin Elmer Corporation, Norwalk, CT, USA). Nuclear magnetic resonance (NMR) spectra, including correlation spectroscopy (COSY), nuclear Overhauser effect spectrometry (NOESY), heteronuclear multiple-bond correlation (HMBC), and heteronuclear single-quantum coherence (HSQC) experiments were acquired using a Varian Unity 400 or a Varian Inova 500 spectrometer (Varian Inc., Palo Alto, CA, USA) operating at 400 and 500 MHz (^1^H) and 100 and 125 MHz (^13^C), respectively, with chemical shifts given in ppm (δ) using tetramethylsilane (TMS) as an internal standard. Electrospray ionization (ESI) and high-resolution electrospray ionization (HR-ESI)-mass spectra were recorded on a Bruker APEX II (Bruker, Bremen, Germany) or a VG Platform Electrospray ESI/MS mass spectrometer (Fison, Villeurbanne, France). Silica gel (70–230, 230–400 mesh, Merck) was used for column chromatography (CC). Silica gel 60 F-254 (Merck, Darmstadt, Germany) was used for thin-layer chromatography (TLC) and preparative thin-layer chromatography (PTLC).

### 3.2. Plant Material

The rhizomes of *C. zedoaria* were collected from Yanpu Township, Pingtung County, Taiwan, in September 2012 and identified by Prof. Jih-Jung Chen. A voucher specimen (CZ-201209) was deposited in the Department of Pharmacy, Tajen University, Pingtung, Taiwan.

### 3.3. Extraction and Isolation

The dried rhizomes (3.2 kg) of *C. zedoaria* were extracted three times with MeOH (12 L each) for 3 days. The extract was concentrated under reduced pressure at 35 °C, and the residue (285 g) was partitioned between EtOAc and H_2_O (1:1) to provide the EtOAc-soluble fraction (fraction A; 98 g). The H_2_O-soluble fraction was further extracted with BuOH, and the BuOH-soluble part (fraction B; 89 g) and the H_2_O-soluble one (fraction C; 95 g) were separated. Fraction A (98 g) was purified by CC (4.4 kg of SiO_2_, 70–230 mesh; *n*-hexane/acetone gradient) to afford 12 fractions: A1–A12. Fraction A3 (7.8 g) was subjected to CC (355 g of SiO_2_, 230–400 mesh; *n*-hexane/acetone 30:1–0:1, 800 mL-fractions) to give 10 subfractions: A3-1–A3-10. Fraction A3-4 (155 mg) was purified by preparative TLC (SiO_2_; CHCl_3_/acetone 50:1) to obtain 1-oleoyl-2,3-distearoylglycerol (**9**) (4.2 mg) (R*_f_* = 0.72). Fraction A3-5 (176 mg) was further purified by preparative TLC (SiO_2_; *n*-hexane/EtOAc 35:1) to obtain germacrone (**4**) (5.2 mg) (*R*_f_ = 0.48). Fraction A3-6 (185 mg) was further purified by preparative TLC (SiO_2_; CHCl_3_/acetone 60:1) to afford curzerenone (**3**) (4.6 mg) (*R*_f_ = 0.69). Fraction A4 (8.1 g) was subjected to CC (365 g of SiO_2_, 230–400 mesh; *n*-hexane/EtOAc 20:1–0:1, 900 mL-fractions) to give 11 subfractions: A4-1–A4-11. Part (154 mg) of fraction A4-2 was further purified by preparative TLC (SiO_2_; *n*-hexane/EtOAc 7:1) to obtain 1-oxocurzerenone (**2**) (4.0 mg). Part (164 mg) of fraction A4-3 was further purified by preparative TLC (SiO_2_; *n*-hexane/acetone 5:1) to obtain curcolone (**5**) (4.5 mg) (*R*_f_ = 0.33). Part (171 mg) of fraction A4-5 was further purified by preparative TLC (SiO_2_; *n*-hexane/EtOAc, 8:1) to yield procurcumenol (**6**) (5.4 mg). Fraction A5 (7.5 g) was subjected to CC (340 g of SiO_2_, 230–400 mesh; CHCl_3_/acetone 15:1–0:1, 950 mL-fractions) to afford nine subfractions: A5-1–A5-9. Fraction A5-4 (365 mg) was purified by MPLC (silica column, CHCl_3_/acetone, 10:1–0:1) to afford six subfractions (each 160 mL, A5-4-1–A5-4-6). Fraction A5-4-3 (52 mg) was purified by preparative TLC (silica gel, CH_2_Cl_2_/acetone, 30:1) to obtain 13-hydroxycurzerenone (**1**) (3.3 mg). Fraction A5-4-4 (58 mg) was further purified by preparative TLC (SiO_2_; CHCl_3_/acetone, 50:1) to afford a mixture of β-sitosterol (**10**) and stigmasterol (**11**) (14.3 mg) (*R*_f_ = 0.56). Part (146 mg) of fraction A5-6 was further purified by preparative TLC (SiO_2_; *n*-hexane/acetone, 10:1) to obtain a mixture of stigmast-4-en-3,6-dione (**12**) and stigmasta-4,22-dien-3,6-dione (**13**) (11.8 mg) (*R*_f_ = 0.38). Fraction A7 (8.1 g) was subjected to CC (420 g of SiO_2_, 230–400 mesh; CH_2_Cl_2_/acetone 10:1–0:1, 950 mL-fractions) to afford 10 subfractions: A7-1–A7-10. Part (154 mg) of fraction A7-6 was purified by preparative TLC (SiO_2_; *n*-hexane/acetone, 8:1) to obtain a mixture of 6β-hydroxystigmast-4-en-3-one (**14**) and 6β-hydroxystigmasta-4,22-dien-3-one (**15**) (10.5 mg) (*R*_f_ = 0.22). Fraction A7-7 (338 mg) was purified by MPLC (silica column, CHCl_3_/MeOH, 10:1–0:1) to afford eight subfractions (each 110 mL, A7-7-1–A7-7-8). Fraction A7-7-4 (48 mg) was purified by preparative TLC (silica gel, CHCl_3_/acetone, 1:1) to yield ermanin (= 3,4′-dimethoxykaempferol) (**7**) (4.5 mg) (*R*_f_ = 0.78). Fraction A8 (6.9 g) was subjected to CC (315 g of SiO_2_, 230–400 mesh; CH_2_Cl_2_/MeOH 8:1–0:1, 800 mL fractions) to afford 11 subfractions: A8-1–A8-11. Part (127 mg) of fraction A8-2 was further purified by preparative TLC (SiO_2_; CHCl_3_/acetone, 15:1) to yield curcumin (**8**) (4.7 mg) (*R*_f_ = 0.68). Part (136 mg) of fraction A8-3 was further purified by preparative TLC (SiO_2_; CH_2_Cl_2_/acetone, 1:1) to afford ermanin (**7**) (3.5 mg) (*R*_f_ = 0.79).

#### 3.3.1. 13-Hydroxycurzerenone (**1**)

Yellowish oil. ${\left[\alpha \right]}_{\text{D}}^{25}$ = + 1.5° (*c* 0.16, CHCl_3_). UV (MeOH): λ_max_ (log ε) = 272 (3.32) nm. IR (neat): υ_max_ = 3450 (OH), 1678 (C=O) cm^−1^. ^1^H-NMR and ^13^C-NMR data (500/125 MHz, in CDCl_3_) were shown in Table 1. ESI-MS: *m*/*z* = 269 [M + Na]^+^. HR-ESI-MS: *m*/*z* = 269.1157 [M + Na]^+^ (calcd. for C_15_H_18_O_3_Na: 269.1154) (Supplementary Materials).

#### 3.3.2. 1-Oxocurzerenone (**2**)

Yellowish oil. ${\left[\alpha \right]}_{\text{D}}^{25}$ = + 2.1° (*c* 0.14, CHCl_3_). UV (MeOH): λ_max_ (log ε) = 273 (3.41) nm. IR (neat): υ_max_ = 1706 (C=O), 1675 (C=O) cm^−1^. ^1^H-NMR and ^13^C-NMR data (500/125 MHz, in CDCl_3_) were shown in Table 1. ESI-MS: *m*/*z* = 269 [M + Na]^+^. HR-ESI-MS: *m*/*z* = 269.1153 [M + Na]^+^ (calcd. for C_15_H_18_O_3_Na: 269.1154) (Supplementary Materials).

#### 3.3.3. Curzerenone (**3**)

Yellowish oil. UV (MeOH): λ_max_ (log ε) = 272 (3.30) nm. IR (neat): υ_max_ = 1676 (C=O) cm^−1^. ^1^H-NMR (CDCl_3_, 500 MHz): δ = 1.19 (3H, s, H-14), 1.84 (3H, s, H-15), 2.19 (3H, d, *J* = 1.5 Hz, H-13), 2.79 (1H, d, *J* = 17.5 Hz, H-9α), 2.91 (1H, d, *J* = 17.5 Hz, H-9β), 3.01 (1H, s, H-5), 4.76 (1H, s, H-3), 4.96 (1H, d, *J* = 17.5 Hz, H-2), 4.97 (1H, d, *J* = 11.0 Hz, H-2), 5.01 (1H, s, H-3), 5.81 (1H, dd, *J* = 17.5, 11.0 Hz, H-1), 7.09 (1H, s, H-12). ESI-MS: *m*/*z* = 231 [M + H]^+^.

#### 3.3.4. Germacrone (**4**)

Colorless crystals (*n*-hexane), mp. 53–55 °C. UV (MeOH): λ_max_ (log ε) = 272 (3.30) nm. IR (KBr): υ_max_ = 1676 (C=O) cm^−1^. ^1^H-NMR (CDCl_3_, 500 MHz): δ = 1.19 (3H, s, H-14), 1.84 (3H, s, H-15), 2.19 (3H, d, *J* = 1.5 Hz, H-13), 2.79 (1H, d, *J* = 17.5 Hz, H-9α), 2.91 (1H, d, *J* = 17.5 Hz, H-9β), 3.01 (1H, s, H-5), 4.76 (1H, s, H-3), 4.96 (1H, d, *J* = 17.5 Hz, H-2), 4.97 (1H, d, *J* = 11.0 Hz, H-2), 5.01 (1H, s, H-3), 5.81 (1H, dd, *J* = 17.5, 11.0 Hz, H-1), 7.09 (1H, s, H-12). ESI-MS: *m*/*z* = 231 [M + H]^+^.

#### 3.3.5. Curcolone (**5**)

Colorless crystals (*n*-hexane-EtOAc), mp. 151–153 °C. ${\left[\alpha \right]}_{\text{D}}^{25}$ = + 13.9° (*c* 0.13, CHCl_3_). UV (MeOH): λ_max_ (log ε) = 214 (3.85), 259 (3.38), 286 (3.37) nm. IR (KBr): υ_max_ = 3445 (OH), 1651 (C=O) cm^−1^. ^1^H-NMR (CDCl_3_, 500 MHz): δ = 1.13 (3H, s, H-14), 1.70-1.81 (2H, m, H-2), 2.05 (3H, s, H-15), 2.24 (3H, d, *J* = 1.5 Hz, H-13), 2.30-2.35 (2H, m, H-3), 2.77 (1H, d, *J* = 17.0 Hz, H-9), 3.11 (1H, d, *J* = 17.0 Hz, H-9), 3.82 (1H, dd, *J* = 10.8, 3.8 Hz, H-1), 7.07 (1H, s, H-12). ESI-MS: *m*/*z* = 269 [M + Na]^+^.

#### 3.3.6. Procurcumenol (**6**)

Viscous oil. ${\left[\alpha \right]}_{\text{D}}^{24}$ = + 218.5° (*c* 0.15, CHCl_3_). UV (MeOH): λ_max_ (log ε) = 248 (3.90), 275 (3.75) nm. IR (neat): υ_max_ = 3430 (OH), 1646 (C=O) cm^−1^. ^1^H-NMR (CDCl_3_, 500 MHz): δ = 1.24 (3H, s, H-14), 1.75 (3H, s, H-13), 1.78 (3H, s, H-12), 1.88 (3H, s, H-15), 2.18 (1H, dd, *J* = 16.0, 13.0 Hz, H-6α), 2.38 (1H, ddd, *J* = 10.5, 10.0, 10.0 Hz, H-1), 2.61 (1H, br d, *J* = 16.0 Hz, H-6β), 5.88 (1H, br s, H-9). ESI-MS: *m*/*z* = 235 [M + H]^+^.

#### 3.3.7. Ermanin (= 3,4′-Dimethoxykaempferol) (**7**)

Yellowish needles (EtOAc), mp. 233–235 °C. UV (MeOH): λ_max_ = 266, 296 (sh), 345 nm. IR (KBr): υ_max_ = 3990 (OH), 1657 (C=O) cm^−1^. ^1^H-NMR (CDCl_3_, 500 MHz): δ = 3.86 (3H, s, OMe-3), 3.90 (3H, s, OMe-4′), 6.28 (1H, d, *J* = 2.0 Hz, H-6), 6.41 (1H, d, *J* = 2.0 Hz, H-8), 7.03 (2H, d, *J* = 9.0 Hz, H-3′ and H-5′), 8.07 (2H, d, *J* = 9.0 Hz, H-2′ and H-6′), 12.73 (1H, s, D_2_O exchangeable, OH-5). ESI-MS: *m*/*z* = 315 [M + H]^+^.

#### 3.3.8. Curcumin (**8**)

Orange crystals (CH_2_Cl_2_-MeOH), mp. 182–183 °C. UV (MeOH): λ_max_ (log ε) = 422 (4.51) nm. IR (KBr): υ_max_ = 3417 (OH), 1629 (C=O) cm^−1^. ^1^H-NMR (CDCl_3_, 400 MHz): δ = 3.96 (6H, s, OMe-3′ and OMe-3′′), 5.81 (1H, s, H-4, this proton signal assignable to an enol form), 5.86 (2H, br s, OH-4′ and OH-4′′), 6.48 (2H, d, *J* = 15.6 Hz, H-2 and H-6), 6.94 (2H, d, *J* = 8.0 Hz, H-5′ and H-5′′), 7.06 (2H, d, *J* = 2.0 Hz, H-2′ and H-2′′), 7.13 (2H, dd, *J* = 8.0, 2.0 Hz, H-6′ and H-6′′), 7.60 (2H, d, *J* = 15.6 Hz, H-1 and H-7). ESI-MS: *m*/*z* = 369 [M + H]^+^.

#### 3.3.9. 1-Oleoyl-2,3-distearoylglycerol (**9**)

Colorless oil. ${\left[\alpha \right]}_{\text{D}}^{25}$ = +3.6° (*c* 0.2, CHCl_3_). IR (neat): υ_max_ = 1744 (C=O) cm^−1^. ^1^H-NMR (CDCl_3_, 500 MHz): δ = 0.88 (9H, t, *J* = 7.0 Hz, H-18′, H-18′′, and H-18′′′), 1.26 (76H, br s, H-4′~H-7′ and H-12′~H-17′, H-4′′~H-17′′, and H-4′′′~H-17′′′), 1.61 (6H, m, H-3′, H-3′′, and H-3′′′), 2.01 (4H, m, H-8′ and H-11′), 2.31 (6H, t, *J* = 7.5 Hz, H-2′, H-2′′, and H-2′′′), 4.15 (2H, dd, *J* = 12.0, 5.5 Hz, H-1a and H-3a), 4.30 (2H, dd, *J* = 12.0, 4.0 Hz, H-1b and H-3b), 5.27 (1H, m, H-2), 5.35 (2H, m, H-9′ and H-10′). ESI-MS: *m*/*z* = 911 [M + Na]^+^.

#### 3.3.10. Mixture of β-Sitosterol (**10**) and Stigmasterol (**11**) (ratio 4:1)

Colorless needles (MeOH), mp. 133–135 °C. ${\left[\alpha \right]}_{\text{D}}^{24}$ = − 35.6° (*c* 0.24, CHCl_3_). IR (KBr): υ_max_ = 3440 (OH) cm^−1^. ^1^H-NMR (CDCl_3_, 500 MHz) of **10**: δ = 0.68 (3H, s, H-18), 0.81 (3H, d, *J* = 6.8 Hz, H-27), 0.83 (3H, d, *J* = 6.8 Hz, H-26), 0.85 (3H, t, *J* = 7.2 Hz, H-29), 0.92 (3H, d, *J* = 6.5 Hz, H-21), 1.01 (3H, s, H-19), 3.52 (1H, m, H-3), 5.35 (1H, d, *J* = 5.5 Hz, H-6); ^1^H-NMR (CDCl_3_, 500 MHz) of **11**: δ = 0.70 (3H, s, H-18), 0.79 (3H, d, *J* = 6.8 Hz, H-27), 0.83 (3H, d, *J* = 6.8 Hz, H-26), 0.82 (3H, t, *J* = 7.2 Hz, H-29), 1.01 (3H, s, H-19), 1.02 (3H, d, *J* = 6.5 Hz, H-21), 3.52 (1H, m, H-3), 5.01 (1H, dd, *J* = 15.5, 8.5 Hz, H-23), 5.15 (1H, dd, *J* = 15.5, 8.5 Hz, H-22), 5.35 (1H, br d, *J* = 5.5 Hz, H-6). ESI-MS of **10**: *m*/*z* = 415 [M + H]^+^; ESI-MS of **11**: *m/z* = 413 [M + H]^+^.

#### 3.3.11. Mixture of Stigmast-4-en-3,6-dione (**12**) and Stigmasta-4,22-dien-3,6-dione (**13**) (ratio 4.7:1)

Light-yellow needles (MeOH), mp. 172–174 °C. ${\left[\alpha \right]}_{\text{D}}^{24}$ = − 38.2° (*c* 0.24, CHCl_3_). UV (MeOH): λ_max_ (log ε) = 250 (4.16) nm. IR (KBr): υ_max_ = 1689 (C=O) cm^−1^. ^1^H-NMR (CDCl_3_, 500 MHz) of **12**: δ = 0.72 (3H, s, H-18), 0.81 (3H, d, *J* = 7.0 Hz, H-27), 0.83 (3H, d, *J* = 7.0 Hz, H-26), 0.86 (3H, t, *J* = 7.0 Hz, H-29), 0.94 (3H, d, *J* = 6.5 Hz, H-21), 1.17 (3H, s, H-19), 6.17 (1H, s, H-4); ^1^H-NMR (CDCl_3_, 500 MHz) of **13**: δ = 0.74 (3H, s, H-18), 0.79 (3H, d, *J* = 7.0 Hz, H-27), 0.83 (3H, d, *J* = 7.0 Hz, H-26), 0.82 (3H, t, *J* = 7.0 Hz, H-29), 1.17 (3H, s, H-19), 1.04 (3H, d, *J* = 6.5 Hz, H-21), 6.17 (1H, m, H-4), 5.04 (1H, dd, *J* = 15.5, 8.5 Hz, H-23), 5.15 (1H, dd, *J* = 15.5, 8.5 Hz, H-22). ESI-MS of **12**: *m*/*z* = 449 [M + Na]^+^; ESI-MS of **13**: *m*/*z* = 447 [M + Na]^+^.

#### 3.3.12. Mixture of 6β-Hydroxystigmast-4-en-3-one (**14**) and 6β-Hydroxystigmasta-4,22-dien-3-one (**15**) (ratio 3.3:1)

Colorless needles (MeOH), mp. 207–209 °C. ${\left[\alpha \right]}_{\text{D}}^{24}$ = + 30.2° (*c* 0.15, CHCl_3_). UV (MeOH): λ_max_ (log ε) = 246 (4.13) nm. IR (KBr): υ_max_ = 3430 (OH), 1676 (C=O) cm^−1^. ^1^H-NMR (CDCl_3_, 500 MHz) of **14**: δ = 0.74 (3H, s, H-18), 0.81 (3H, d, *J* = 7.0 Hz, H-27), 0.84 (3H, d, *J* = 7.0 Hz, H-26), 0.87 (3H, t, *J* = 7.0 Hz, H-29), 0.92 (3H, d, *J* = 6.5 Hz, H-21), 1.38 (3H, s, H-19), 4.35 (1H, t, *J* = 3.0 Hz, H-6), 5.82 (1H, s, H-4); ^1^H-NMR (CDCl_3_, 500 MHz) of **15**: δ = 0.76 (3H, s, H-18), 0.80 (3H, d, *J* = 7.0 Hz, H-27), 0.81 (3H, d, *J* = 7.0 Hz, H-26), 0.85 (3H, t, *J* = 7.2 Hz, H-29), 1.38 (3H, s, H-19), 1.02 (3H, d, *J* = 6.0 Hz, H-21), 4.35 (1H, t, *J* = 3.0 Hz, H-6), 5.03 (1H, dd, *J* = 15.0, 8.5 Hz, H-23), 5.15 (1H, dd, *J* = 15.0, 8.5 Hz, H-22), 5.82 (1H, s, H-4). ESI-MS of **14**: *m*/*z* = 429 [M + H]^+^; ESI-MS of **15**: *m*/*z* = 427 [M + H]^+^.

### 3.4. Anti-Platelet Aggregation Test

Blood was collected from the rabbit marginal vein, anticoagulated with EDTA (6 mM) and centrifuged for 10 min at 90× *g* at room temperature to obtain platelet-rich plasma (PRP). Platelet suspension was prepared from this EDTA-anticoagulated PRP according to the washing procedures described previously [20]. Platelet numbers were counted by a Coulter counter (Model ZM) and adjusted to 4.5 × 10^8^ platelets/mL. The platelet pellets were finally suspended in Tyrode's solution of the following composition (mM): NaCl (136.8), KCl (2.8), NaHCO_3_ (11.9), MgCl_2_ (2.1), NaH_2_PO_4_ (0.33), CaCl_2_ (1.0), and glucose (11.2), containing bovine serum albumin (0.35%). Platelet aggregation was measured at 37 °C by the turbidimetric method as described by O’Brien [19] using a Chrono-log Lumi-aggregometer. The platelet suspensions were stirred at 1200 rpm. All the tested compounds were dissolved in dimethyl sulfoxide (DMSO). In order to eliminate the effect of the solvent on the aggregation, the final concentration of DMSO was fixed at 0.5%, and did not affect the aggregation measured. Percentages of aggregation were calculated using the absorbance of platelet suspension to represent 0% aggregation and the absorbance of Tyrode’s solution as 100% aggregation. Aspirin was used as a positive control. Data were analyzed using Student’s *t*-test.

#### Statistical Analysis

Results are expressed as the mean ± SEM, and comparisons were made using Student’s *t*-test. A probability of 0.05 or less was considered significant. The software SigmaPlot (version 11.0, SPSS Inc., Chicago, IL, USA) was used for the statistical analysis.

## 4. Conclusions

Fifteen compounds, including two new sesquiterpenoids, 13-hydroxycurzerenone (**1**) and 1-oxocurzerenone (**2**), were isolated from the rhizomes of *C. zedoaria*. The structures of these compounds were established on the basis of spectroscopic data. The anti-platelet effects of the isolates were evaluated by suppressing collagen- and AA-induced platelet aggregation in washed rabbit platelets. The results of anti-platelet aggregation experiments indicate that compounds **1**, **3**, **5**, **7**, **8**, and the mixture of **12** and **13** can significantly inhibit collagen- and/or AA-induced platelet aggregation at 100 μM. Ermanin (**7**) and curcumin (**8**) were the most effective among the isolated compounds, with inhibition % of 67.58 ± 3.82 and 95.36 ± 1.58 μg/mL, respectively, against collagen- and AA-induced platelet aggregation at 100 μM. Our study suggests that *C. zedoaria* and its isolates (especially **3**, **7**, and **8**) could be further developed as potential candidates for the treatment or prevention of cardiovascular diseases.

## Figures and Tables

**Table 1:** ^1^H- and ^13^C-NMR data of **1**–**3**.

Position	1 ^a^		2 ^a^		3 ^b^	
	δ_H_	δ_C_	δ_H_	δ_C_	δ_H_	δ_C_
1	5.82 dd (17.5, 11.0)	145.5		212.1	5.78 dd (17.6, 10.6)	145.5
2	4.96 d (17.5)	115.6	2.13 s	25.6	4.74–4.99 m	115.7
	4.97 d (11.0)					
3	4.76 s	113.0	4.75 s	113.0	4.74–4.99 m	113.0
	5.01 s		5.01 s			
4		141.1		141.0		141.1
5	3.02 s	64.1	3.22 s	61.9	3.00 s	64.1
6		194.0		194.0		194.0
7		120.2		120.1		120.2
8		165.6		165.6		165.6
9α	2.79 d (17.5)	33.6	2.94 d (17.5)	28.5	2.83 d (17.6)	33.6
9β	2.91 d (17.5)		3.06 d (17.5)		2.83 d (17.6)	
10		42.9		46.2		42.9
11		116.0		119.3		119.3
12	7.09 s	139.6	7.08 s	139.6	7.08 br s	139.6
13	4.86 s	52.2	2.18 d (1.5)	9.0	2.16 br s	9.1
14	1.19 s	25.0	1.18 s	16.2	1.17 s	25.0
15	1.84 s	24.9	1.83 s	24.7	1.83 br s	24.9

^a^ Recorded in CDCl_3_ at 500 MHz (^1^H) and 125 MHz (^13^C). ^b^ Recorded in CDCl_3_ at 300 MHz (^1^H) and 75 MHz (^13^C) [6]. Values in ppm (δ). *J* (in Hz) in parentheses.

**Table 2:** Inhibitory effects of compounds on the aggregation of washed rabbit platelets induced by collagen and arachidonic acid (AA) ^a^.

Compounds (100 μM)	Collagen (10 μg/mL)	Arachidonic Acid (200 μM)
	Inhibition (%) ^a,b^	
13-Hydroxycurzerenone (**1**)	22.82 ± 0.98 ^d^	31.65 ± 5.33 ^c^
1-Oxocurzerenone (**2**)	23.22 ± 0.68 ^d^	12.55 ± 1.59
Curzerenone (**3**)	46.11 ± 0.38 ^e^	15.41 ± 1.24 ^c^
Germacrone (**4**)	21.97 ± 1.15 ^d^	13.22 ± 2.18
Curcolone (**5**)	35.85 ± 0.75 ^c^	23.44 ± 3.35 ^c^
Procurcumenol (**6**)	21.28 ± 1.76 ^d^	11.02 ± 0.86
Ermanin (**7**)	67.58 ± 3.82 ^e^	34.37 ± 2.74 ^e^
Curcumin (**8**)	31.57 ± 2.10 ^e^	95.36 ± 1.58 ^e^
1-Oleoyl-2,3-distearoylglycerol (**9**)	4.10 ± 0.77	3.97 ± 0.27
Mixture of β-sitosterol (**10**) and stigmasterol (**11**)	3.08 ± 0.43	4.13 ± 0.64
Mixture of stigmast-4-en-3,6-dione (**12**) and stigmasta-4,22-dien-3,6-dione (**13**)	42.71 ± 2.11 ^c^	23.92 ± 2.31 ^d^
Mixture of 6β-hydroxystigmast-4-en-3-one (**14**) and 6β-hydroxystigmasta-4,22-dien-3-one (**15**)	8.21 ± 1.06	7.53 ± 0.95
Aspirin ^b^	5.20 ± 0.35 ^e^	100.0 ± 0.0 ^e^

^a^ Platelets were preincubated with each compound or dimethyl sulfoxide (DMSO) (0.5%, control) at 37 °C for 3 min, then the inducer collagen or arachidonic acid (AA) was added. Results are presented as averages ± SEM (*n* = 3–4). ^b^ Aspirin was used as the positive control. ^c^ *p* < 0.05; ^d^ *p* < 0.01; ^e^ *p* < 0.001 compared to control.

## Supplementary Materials

Supplementary materials can be found at www.mdpi.com/1420-3049/21/10/1385/s1.

## Abbreviations

fMLP	*N*-formyl-L-methionyl-L-leucyl-L-phenylalanine
CB	cytochalasin B
COSY	correlation spectroscopy
NOESY	nuclear Overhauser effect spectrometry
HMBC	heteronuclear multiple-bond correlation
HSQC	heteronuclear single-quantum coherence
DEPT	distortionless enhancement by polarization transfer
ESI	electrospray ionisation
HRESI	high-resolution electrospray ionization
CC	column chromatography
TLC	thin-layer chromatography
MPLC	medium pressure liquid chromatography
