# Supplementary material for: New Sesquiterpenoids and Anti-Platelet Aggregation Constituents from the Rhizomes of Curcuma zedoaria
Source: Molecules. 2016 Oct 17;21(10):1385. doi: 10.3390/molecules21101385 (PMC6272984; doi:10.3390/molecules21101385)
Supplement: Supplementary file 1 [file molecules-21-01385-s001.pdf]

Supplementary Materials: New Sesquiterpenoids and Anti-Platelet Aggregation Constituents from the Rhizomes of *Curcuma zedoaria*

Jih-Jung Chen, Tung-Han Tsai, Hsiang-Ruei Liao, Li-Chai Chen, Yueh-Hsiung Kuo, Ping-Jyun Sung, Chun-Lin Chen and Chun-Sheng Wei

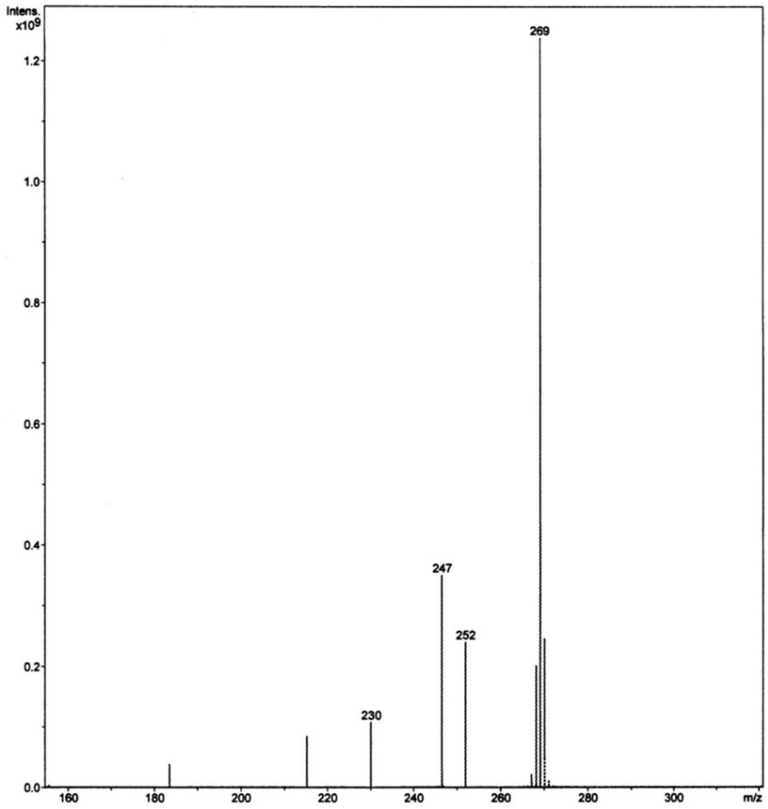

Figure S1. ESI-MS spectrum of **1**.

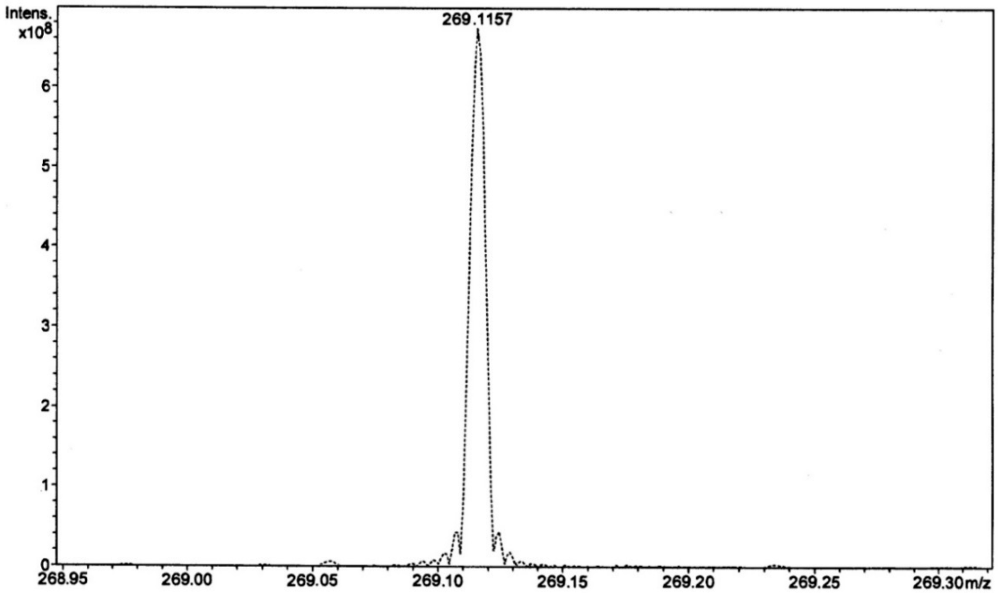

Figure S2. HR-ESI-MS spectrum of **1**.

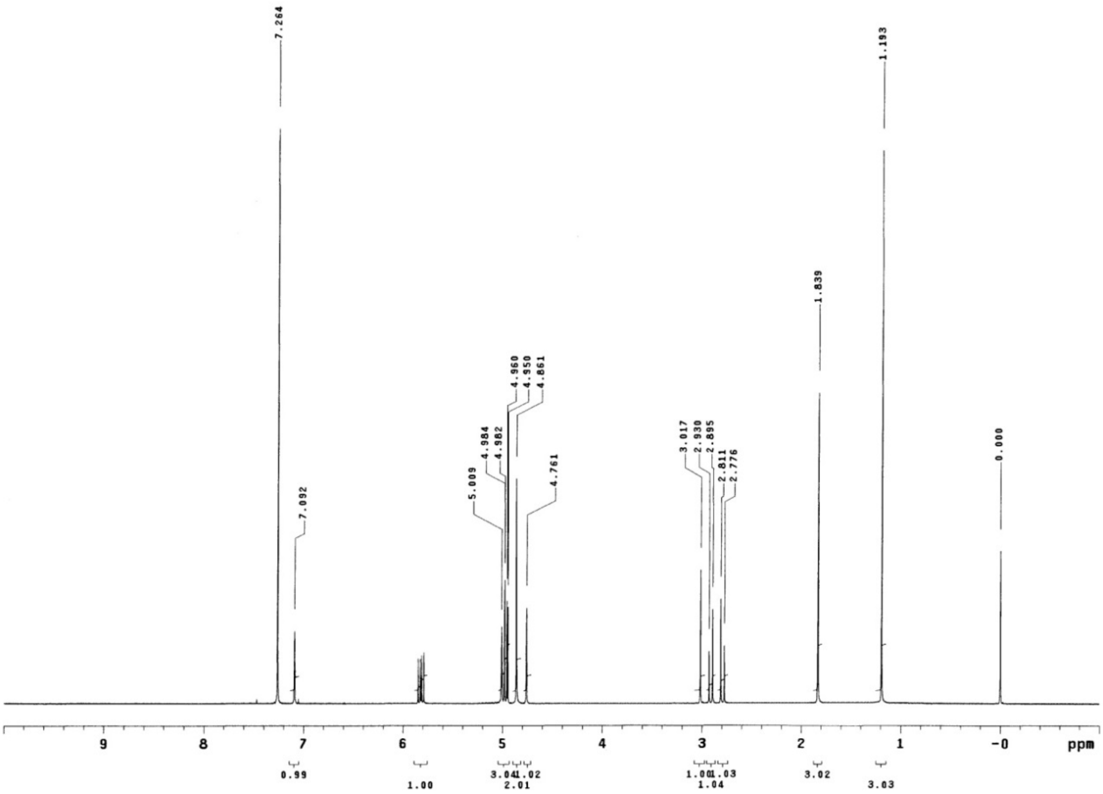

Figure S3. ^{1}H-NMR spectrum of **1** ($CDCl_3$, 500 MHz).

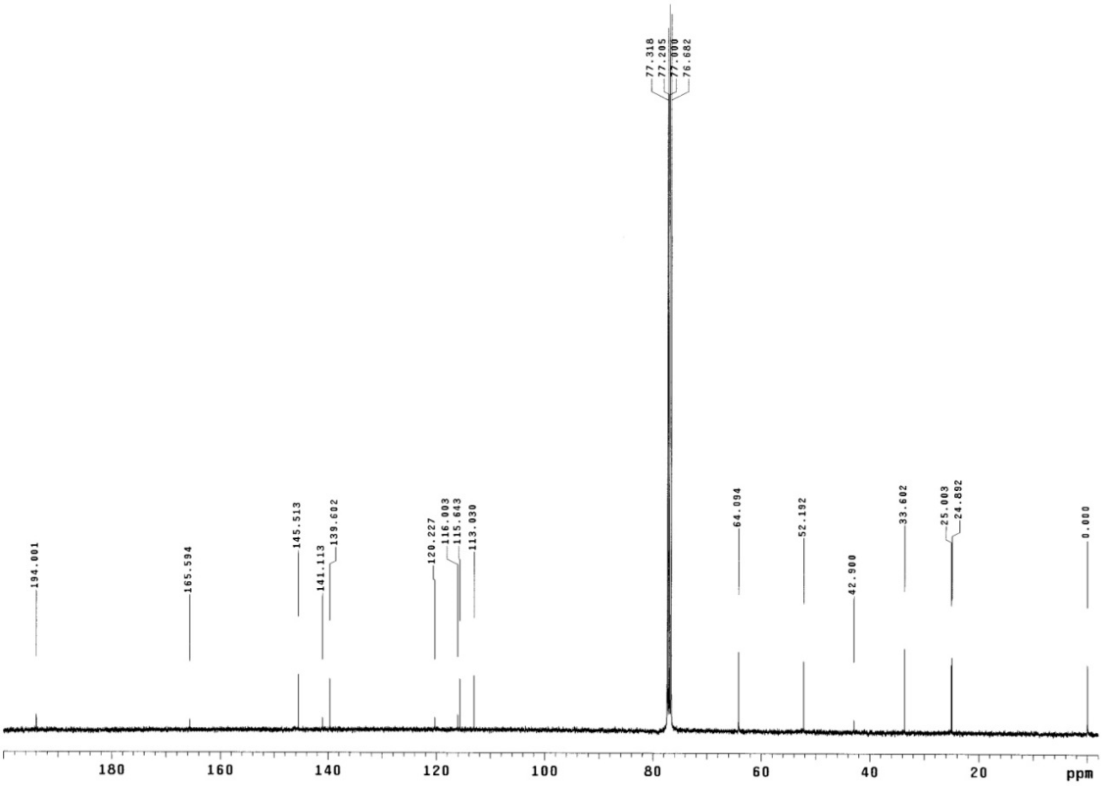

Figure S4. ^{13}C-NMR spectrum of **1** ($CDCl_3$, 125 MHz).

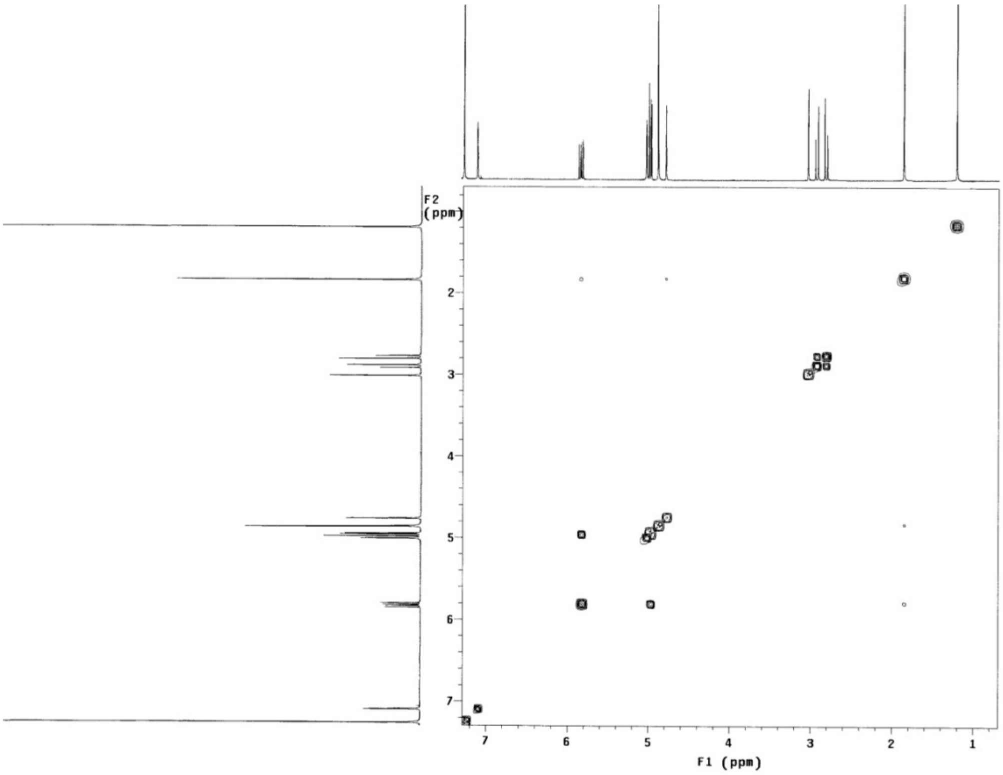

Figure S5. ^1H–^1H COSY spectrum of **1**.

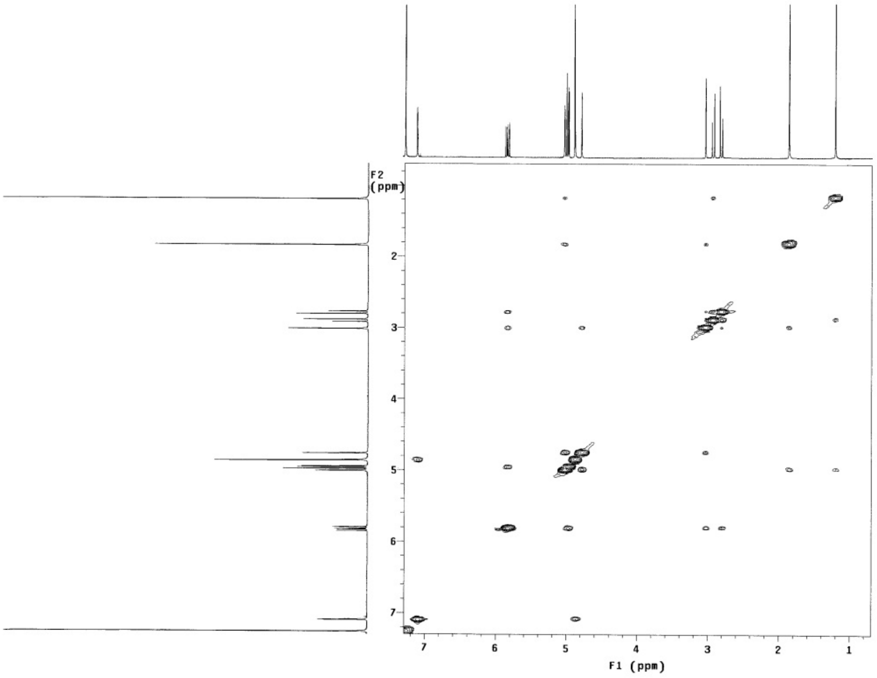

Figure S6. NOESY spectrum of **1**.

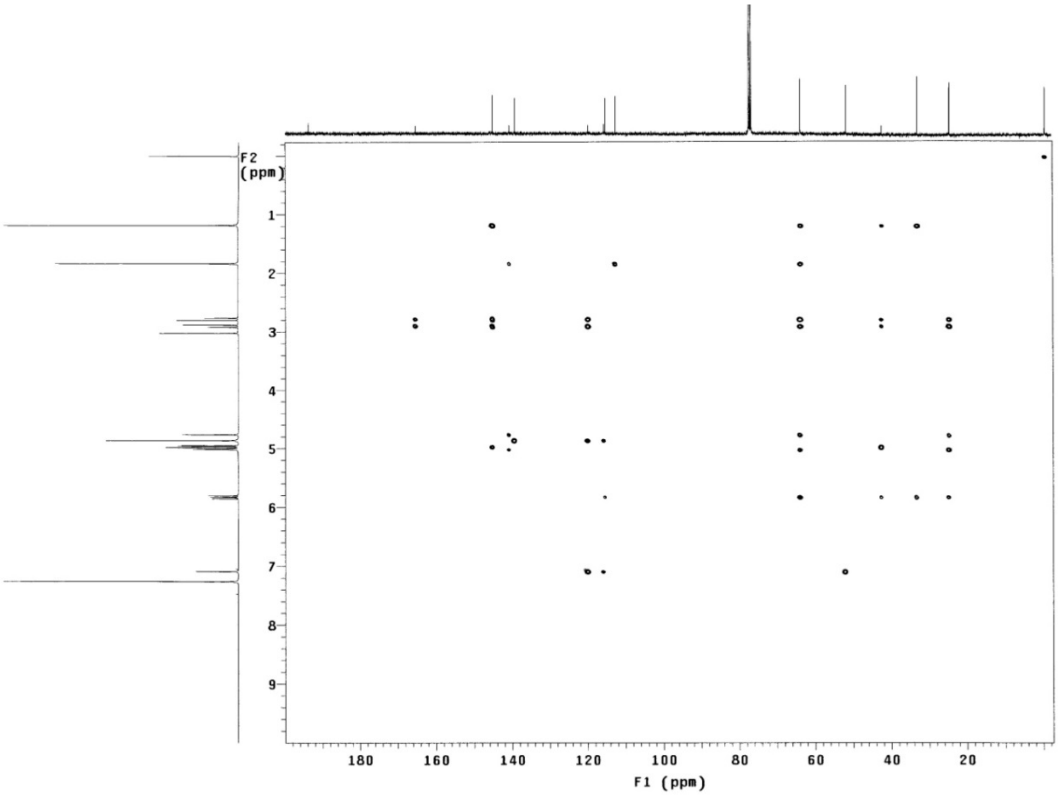

Figure S7. HMBC spectrum of **1**.

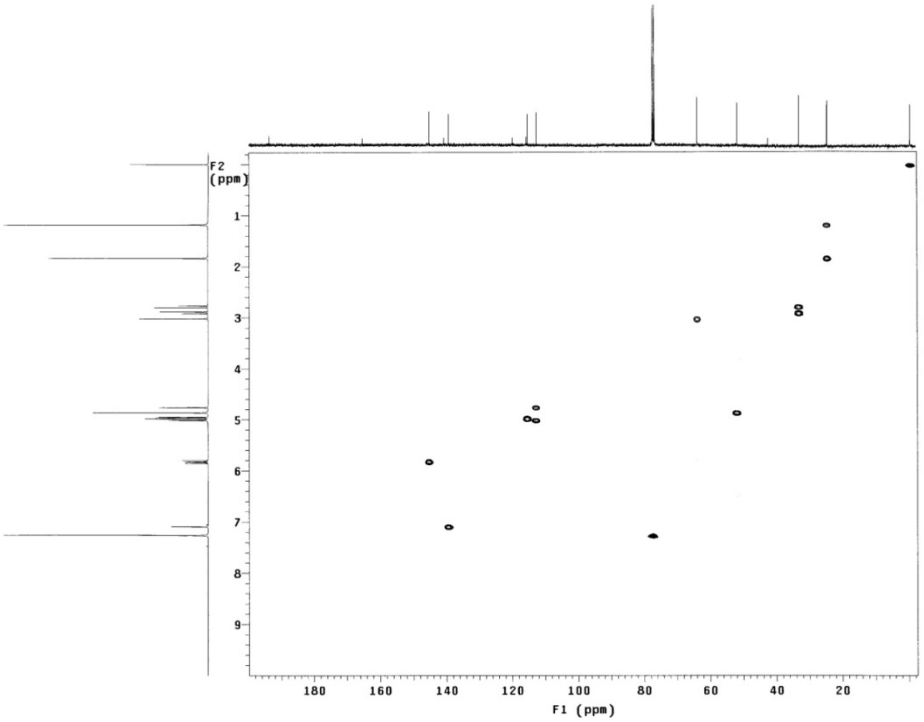

Figure S8. HSQC spectrum of **1**.

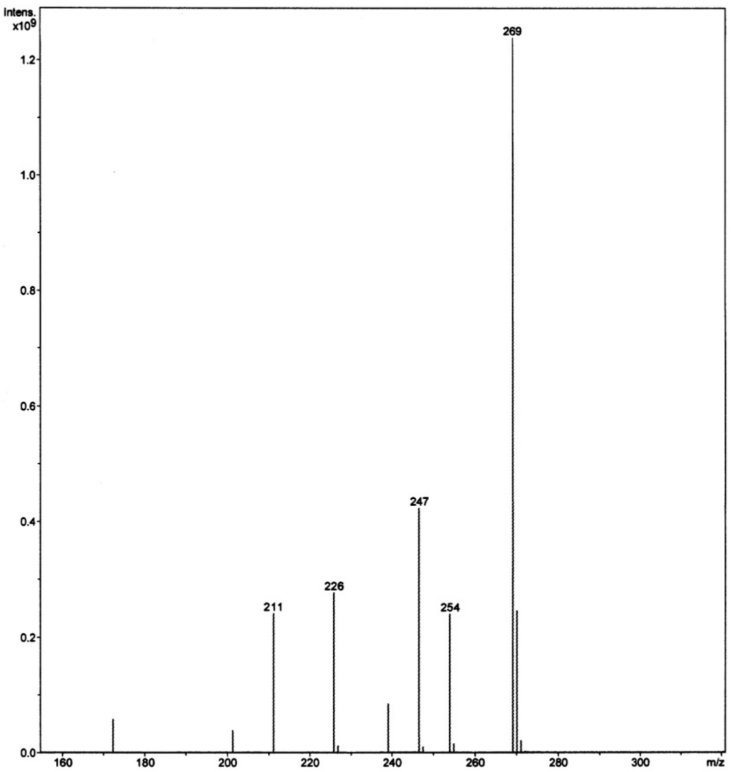

Figure S9. ESI-MS spectrum of **2**.

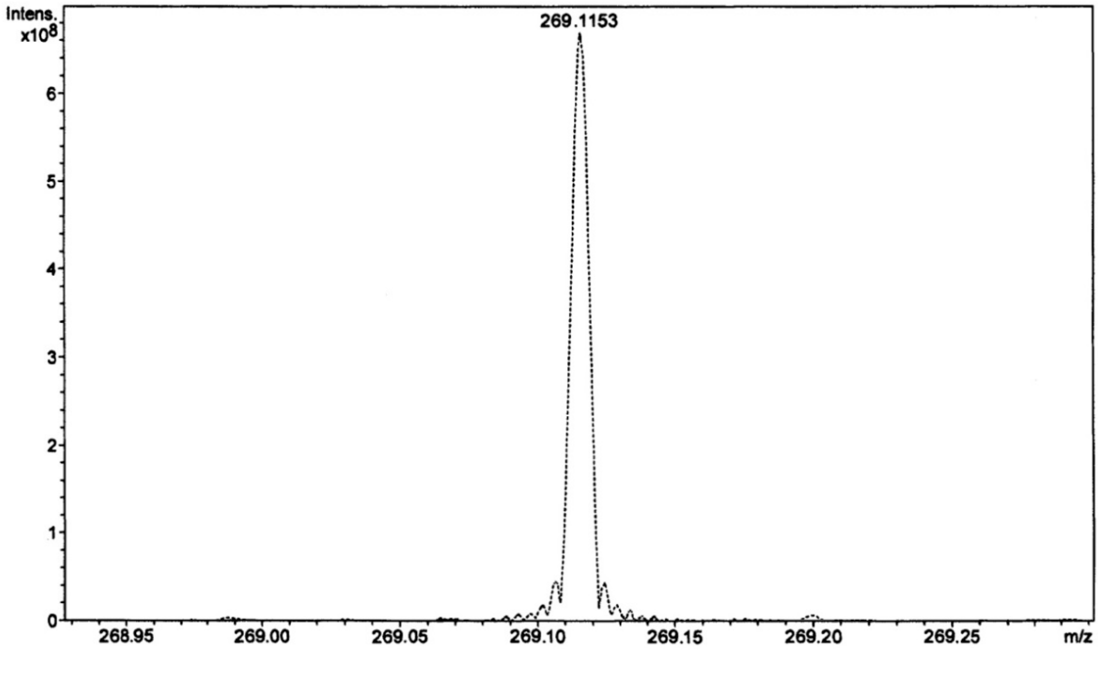

Figure S10. HR-ESI-MS spectrum of **2**.

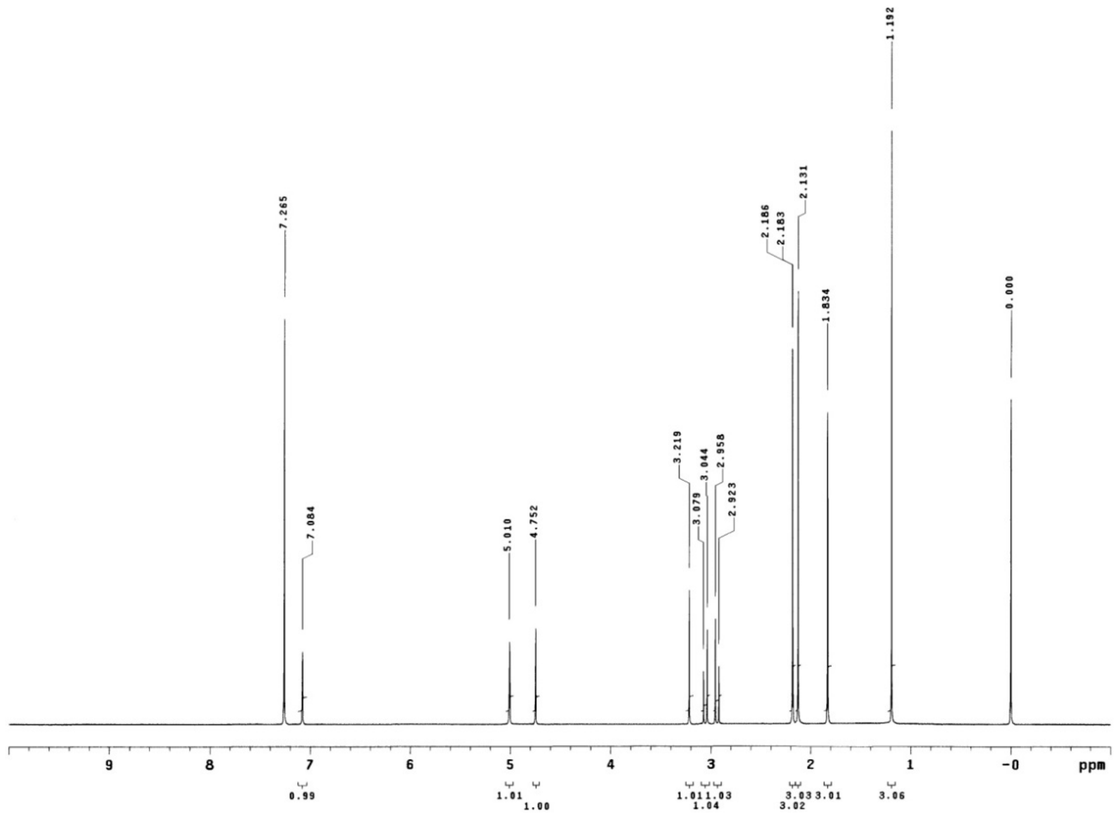

Figure S11. ^{1}H-NMR spectrum of **2** ($CDCl_3$, 500 MHz).

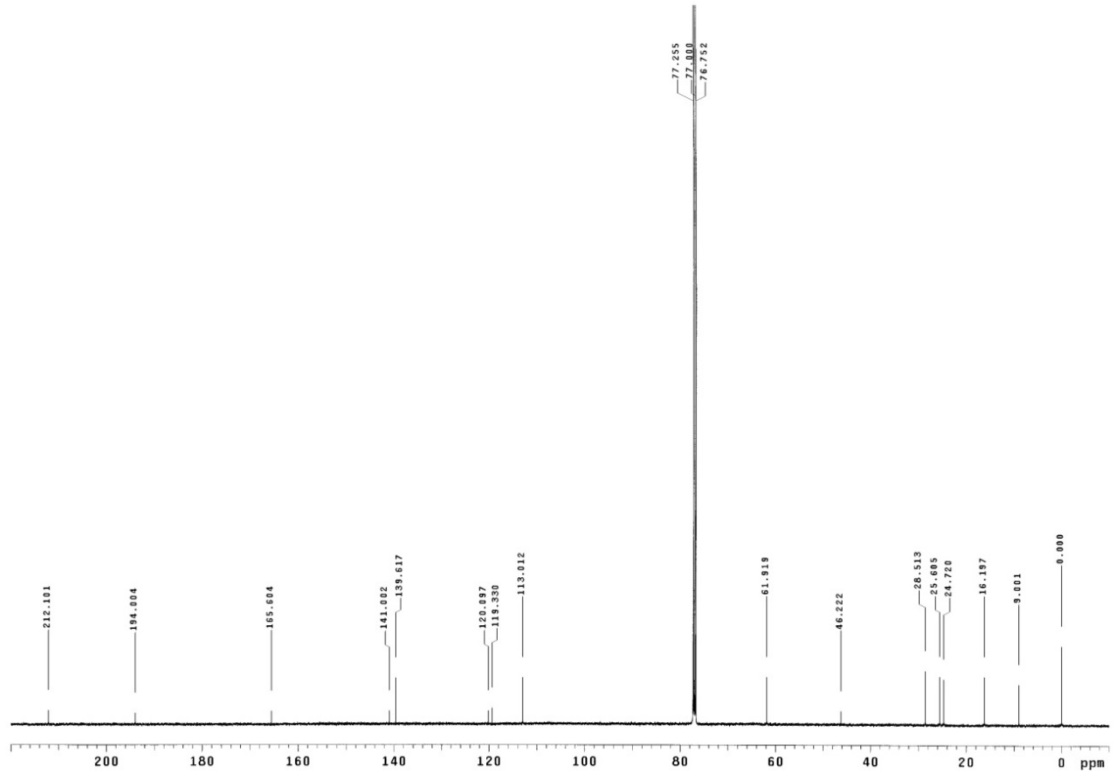

Figure S12. ^{13}C-NMR spectrum of **2** ($CDCl_3$, 125 MHz).

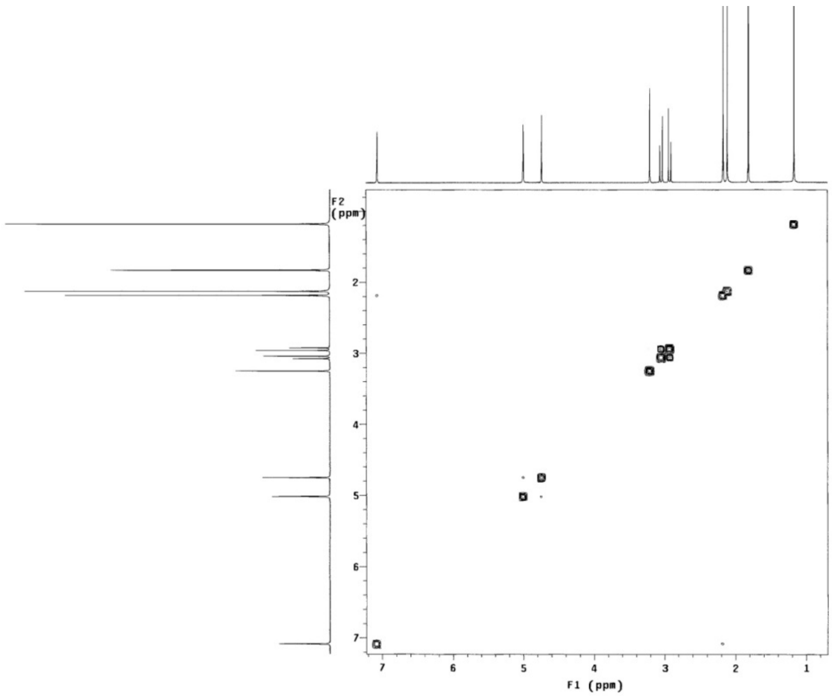

Figure S13. $^{1}H–^{1}H$ COSY spectrum of **2**.

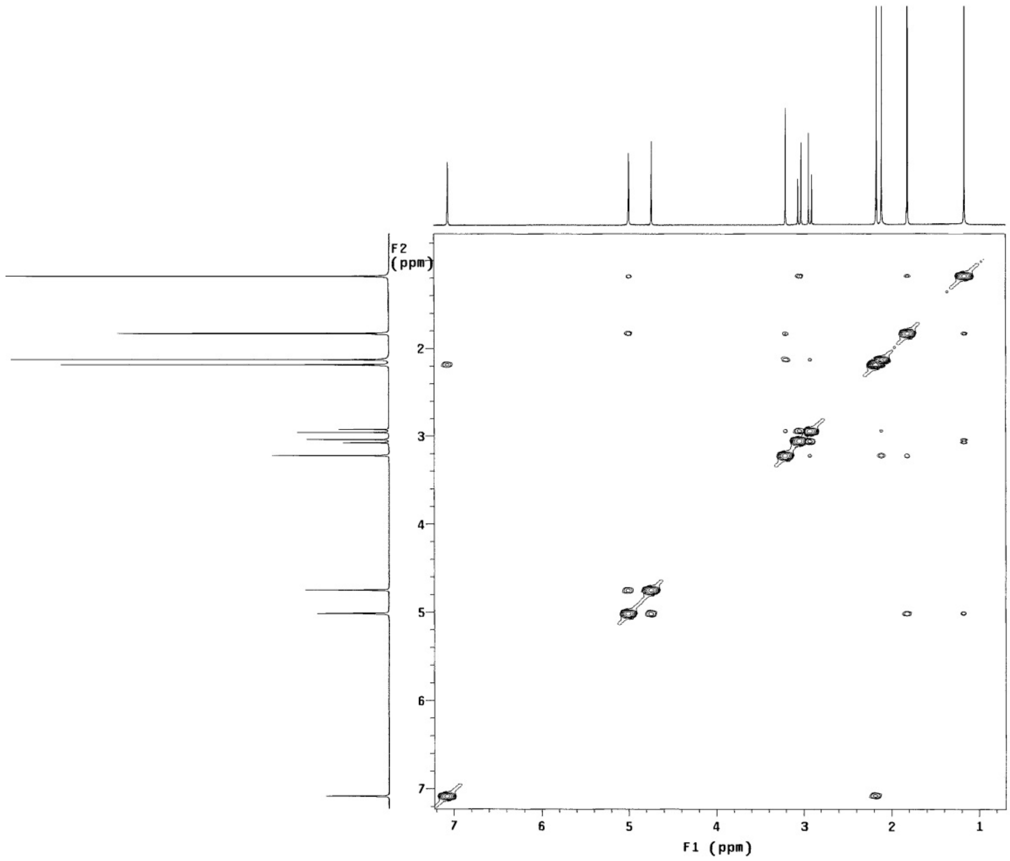

Figure S14. NOESY spectrum of **2**.

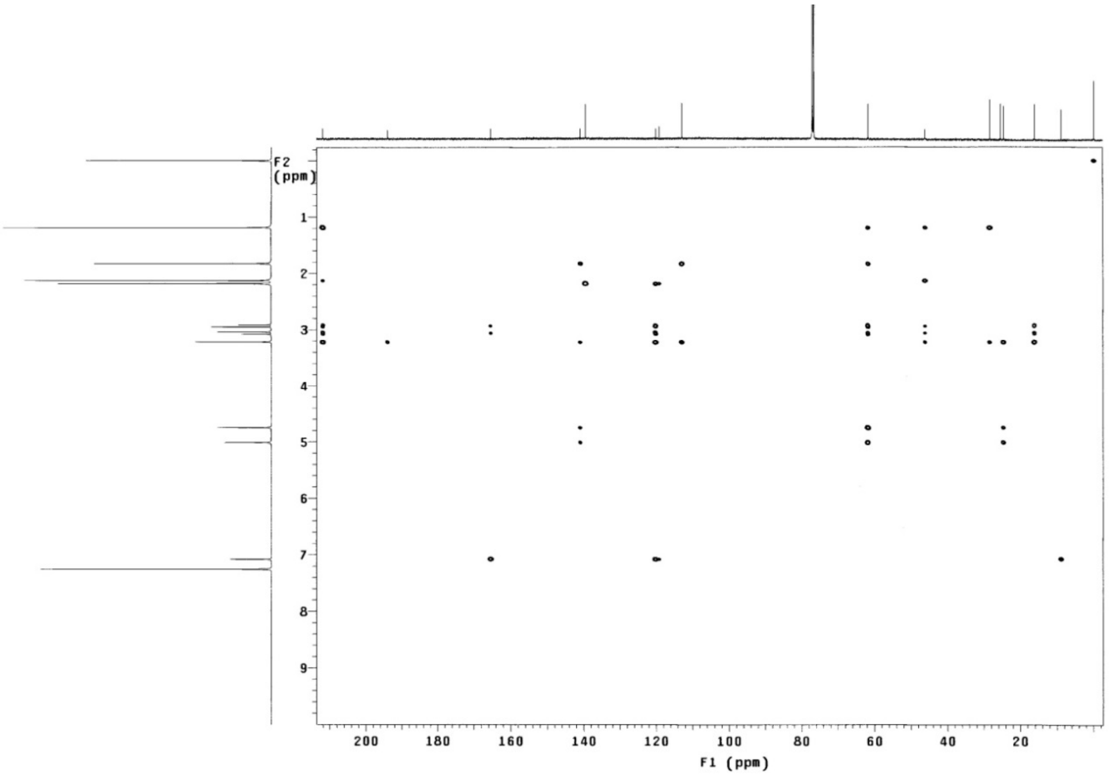

Figure S15. HMBC spectrum of **2**.

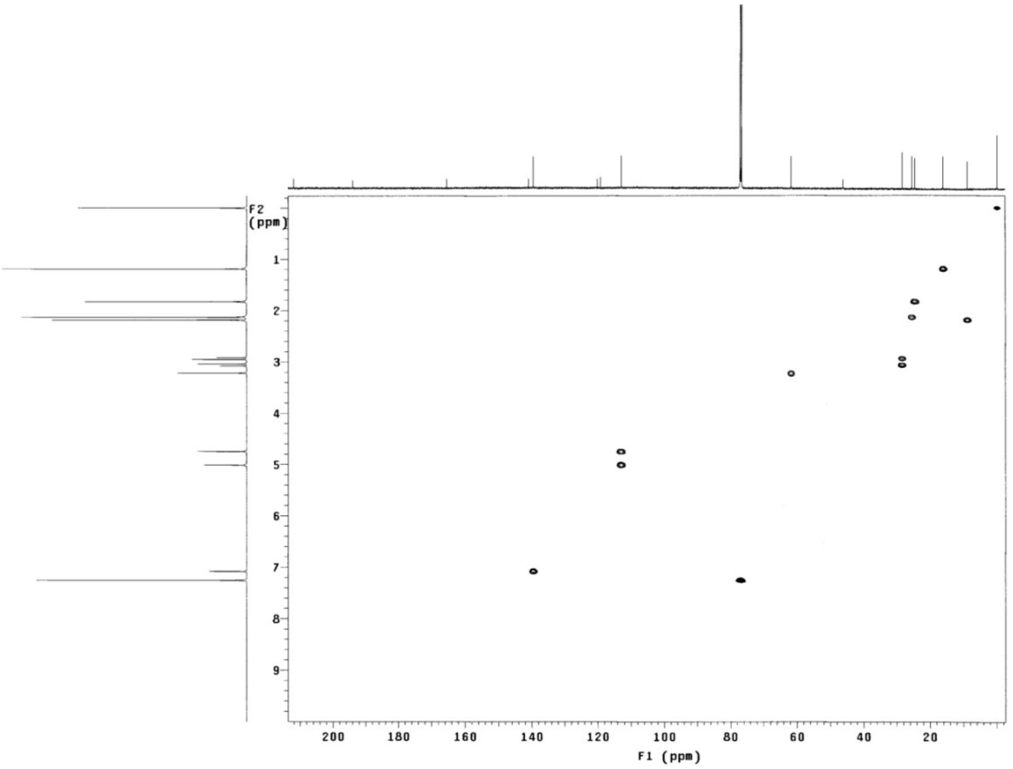

Figure S16. HSQC spectrum of **2**.
